# Assessing the Feasibility of Adapting a School-Based HIV Prevention Intervention to Include Voluntary Male Medical Circumcision and Vaccination for Human Papilloma Virus Prevention

**DOI:** 10.24248/eahrj.v4i1.628

**Published:** 2020-06-26

**Authors:** Burmen Barbara, Obunga Joseph, Omollo Mevis, Kennedy Mutai

**Affiliations:** a Kenya Medical Research Institute Centre for Global Health Research, Kisumu, Kenya

## Abstract

In 2012, there were 2,454 cases and 1,676 deaths from cervical cancer in Kenya. Human Papilloma Virus (HPV) is responsible for 99% of all cervical cancers. National cervical cancer prevention guidelines recommend HPV vaccination among HPV-naïve pre-adolescent girls' prior to onset of sexual activity preferably through school-based interventions. Similarly, Voluntary Male Medical Circumcision (VMMC) programs also reduce sexually transmitted infections like HIV, and ideally should also be conducted prior to the onset of sexual activity. The Families Matter! Program (FMP) is a school-based evidence-based HIV prevention intervention for parents and guardians of pre-adolescents aimed to enhance protective parenting practices in order to reduced sexual risk-taking among adolescents. In this paper we describe how we will recruit a cohort of 55 parent-child dyads in a primary school in Kisumu County then implement and evaluate an adapted FMP program that incorporates VMMC promotion and HPV vaccination in conjunction. It is anticipated that the intervention will enhance parental-child communication about sexual matters, promote safe sexual practices and uptake of biomedical prevention interventions and overall reproductive health among the pre-adolescents

## BACKGROUND

Cervical cancer is the second most common cancer in women living in resource limited settings. There were approximately 445 000 new cases in 2012 in resource limited settings contributing to 84% of the new cases worldwide. In 2012, approximately 270 000 women died from cervical cancer; more than 85% of these deaths occurring in low- and middle-income countries.^1^ Among patients with cancer, the survival rates of those in sub Saharan Africa is 21% compared to 70% in the US.^2^ The Nairobi Cancer registry documented that between 2004 and 2008, there were 8,982 cases of cancer in Nairobi with 5,093 among women. Cervical cancer contributed to 21% of the cancer cases among women.^3^ In Kenya in 2012, there were 2,454 cases of cervical cancer cases, 1,676 cancer deaths and a cervical cancer screening coverage rate of 3.2% among women aged 18-69 years.^4, 5^ Human Papilloma Virus (HPV) is responsible for 99% of all cervical cancers. HPV subtypes 16 and 18 cause up to 70% of all cancers.^1^ Human Immunodeficiency Virus (HIV) is postulated to facilitate the progression of HPV infection to cervical cancer.^6^ The prevalence of cervical cancer is therefore unsurprisingly highest in developing countries which also bear the burden of HIV.^1, 7^

Cervical cancer can be prevented through HPV vaccination among pre-pubescent girls. The Kenya cervical cancer prevention guidelines recommend the administration of HPV vaccine to pre/early adolescent prior to onset of sexual activity. This should preferably be done through a school-based program targeting girls in primary school from classes 4-8, corresponding to an age range of 9 to 13 years. The rationale for this age group is that they are HPV-naïve and will have the highest antibody response to the vaccine.^8^ Whilst awareness of cervical cancer has been shown to promote uptake of HPV vaccine, ^9^ barriers exist, and studies suggest that some Kenyan parents of adolescent children fear that that administration of an HPV vaccine could encourage their daughters to have sex. Published literature documents HPV vaccine uptake of 31% and completion of 70%.^9^ Furthermore, the HPV vaccine is not yet available in routine clinical care in Kenya.

Male Circumcision (MC) has also been shown to have a protective effect against Sexually Transmitted Infections (STIs) like HPV and HIV. Male circumcision (MC) reduces the risk of STI and HIV acquisition in men by 35% and 60% respectively.^10, 11^ The Kenya Voluntary Male Medical Circumcision (VMMC) strategy (2014/5-2018), aims to reduce new HIV infections by increasing the proportion of men aged 15-49 that are circumcised to 80%. For the greatest effect in preventing HIV acquisition, MC should be performed prior to the onset of sexual activity.^12^ Published literature documents a VMMC up-take of 71% through school-based programs.^13^ In Kenya sexual activity has been documented to begin early, prior to 15 years of age.^14^ As such, school based interventions have been widely implemented to reach large numbers of adolescents with HIV and SRH messages.^15^

Families Matter! Program (hereafter referred to as FMP) is an evidence-based HIV prevention intervention for parents and guardians of adolescents aged 9-12 years. The exclamation is placed to emphasise the importance of the families in helping adolescents avoid risky behaviour such as early sexual activity and drug abuse. FMP aims to enhance protective parenting practices that are associated with reduced sexual risk-taking among adolescents and promote parent-child communication about sexuality and risk reduction. FMP provides education on HIV prevention, STI risk reduction and contraception. Families Matter! Program was modified to the Kenyan context from the US-based Parents Matter! Program in 2003-2004. Following its implementation, the programme was evaluated and shown to be acceptable and also retained its effectiveness in promoting parental skills, parent-child communication and adolescent sexual risk reduction.^18^ FMP retention rates of 90% have been documented.^17^ During implementation, Kenyan parents reported having felt ill-equipped to discuss sexual matters with their children.^16^

FMP's theoretical framework is guided by four theories. Firstly *the social learning theory*, to encourage parents to provide supportive environments, reinforce competencies, supervise their children and reduce contact with poor role models. Secondly *the problem behaviour theory* to bolster children's competencies, promote positive behaviour and reduce opportunities to engage in risk behaviour. Thirdly the *theory of reasoned action* equips parents with skills to communicate their own attitudes and norms to their adolescents to modify the adolescent's behaviour. Finally, *the social cognitive theory* enhances parents' efficacy in their ability to communicate with their children about sexual topics. ^19^

Nationally interventions to provide HPV vaccination, VMMC and family education are currently provided in isolation.^8, 15, 17^ We propose to provide an adapted FMP program that incorporates VMMC promotion as a component of HIV prevention and HPV vaccination as a component of STI risk reduction among parents/guardians at one public primary school within Kisumu County to enhance HPV prevention. In this paper we describe our methods that we propose to undertake and specific aims we intend to achieve in order to assess the feasibility of implementing an adapted FMP in the Kenyan setting.

## METHODS

### Study Design and Setting

We will conduct a cohort study among parent-child dyads within one public primary day school in Kisumu County.^20^ Kisumu County was chosen based on its high HIV prevalence (16% vs. 5% country wide),^21^ the highest proportion of young persons whose age of sexual debut was before 15 years (21% vs. 12%) and the highest prevalence of STIs (3% vs. 2%) among sexually active persons in the reproductive age group.^21^ Day primary schools provide a suitable target population of children in classes' 4-8 from which to draw children who are in daily contact with their parents to enhance parent-child communication.^18^

### Study Population

The study population will be comprised of parents of at least one pre/early-adolescent male or female child (aged 9-12 years) in day schools. The age range chosen is ideal for each of the three programs i.e. FMP 9-12 years,^18^ HPV vaccination 9-13 years, ^8^ VMMC 10-14 years,^15^ all of which are best delivered before the onset of sexual activities.^14^ The parents must give informed consent, commit to attending all six FMP sessions, bring their child to the 5^th^ session and participate in ‘home assignments’.^18^ Only one eligible child per household will be included in the study. Pre/early adolescent boys that have already undergone VMMC (and subsequently their parents)will be ineligible for inclusion. Similarly, pre/early-adolescent girls that have already received HPV vaccination will also be excluded (along with their parents). Where either parental consent or assent from the pre/early-adolescent cannot be obtained, the parent-child dyad will be ineligible for inclusion.

### Sample Size

To design the evaluation study, we developed a series of specific aims and corresponding hypotheses:

To determine the uptake and completion rate of HPV vaccine and associated parent and child characteristics among pre/early-adolescent girls who participated in an adapted school-based FMP

*We hypothesize that promotion of HPV vaccine uptake through FMP will double vaccine uptake from 31% to 62% while maintaining the same completion rates of 71%*

To determine the uptake of VMMC and associated parent and child characteristics among pre-adolescent boys who participated in a school-based FMP

*We hypothesize that FMP will attain a VMMC uptake of 80% from the inception of the program to the 6 months booster session*

To document lessons learnt during the promotion of HPV vaccination for pre/early-adolescent girls and VMMC for pre/early-adolescent boys through a school-based FMP.

*We hypothesize that the program will provide valuable lessons as to the feasibility and outcome of promotion of HPV vaccine uptake and VMMC through a school-based FMP program*

We based our subsequent sample size computation on HPV vaccine uptake of 31% and completion of 70% ^9^ and VMMC uptake of 71% through school based programs ^13^ as well as and FMP retention rates of 90% ^17^ documented in the literature. Using HPV vaccine uptake (in order to obtain the largest sample size^22^), and sample size formula for calculation of difference in proportions, to double HPV vaccine uptake (from 31% to 62%) with a significance level of 5% and power of 90% would require a sample size of 50.^23^

$$\begin{array}{l} n=\frac{[p_{1}\left(1-p_{1}\right)+p_{2}\left(1-p_{2}\right)]XC_{p,power}}{{\left(p_{1}-p_{2}\right)}^{2}}\\ \text{n}=\frac{[0.62\left(1-0.62\right)+0.31\left(1-0.31\right)]\text{X }10.6}{{\left(0.62-0.31\right)}^{2}}\\ =49.1\\ =50 \end{array}$$

To adjust for an attrition rate of 10%, a total of 55 dyads will be recruited to participate.^20^ Given the FMP recommends groups of 12-18 participants, we will recruit four groups of approximately 15 parent-child dyads. To improve retention rates, we will provide transport reimbursement and refreshments during sessions. Although absenteeism will be discouraged, makeup sessions will be given for parents who miss a session and are willing to continue with other sessions. We will attempt to make-up for missed sessions by having parents attend make-up sessions in alternate groups as this can affect group dynamics.^18^

### Procedures

#### Adaptation of the FMP program:

The current FMP curriculum^18^ will be adapted to include information on VMMC^15^ and HPV vaccination^8^ that is currently found in different HIV program guidelines by a team of health program managers and certified FMP providers.

#### Notification of County Health Management Teams, School Heads and participating health facilities:

The County Health Management Team (CHMT) will be contacted to obtain approval to conduct the study. We will adapt the methods used by a school-based VMMC program in the former Nyanza province when conducting school-based VMMC projects.^15^ The County Health Promotion Officers (under the guidance of the County Director for Health) who will be involved in the study will notify the County Education Officers who will in turn get in touch with the School heads of the potentially participating schools. Once a school is finally chosen, the study team will make an inception visit to plan study implementation with the school head. We will also involve the head of the participating health facility to ensure that the facility is equipped to implement study procedures

#### Recruitment of potential participants:

During a Parents-Teachers meeting, a study team member will make a short presentation about the study. Interested parents/guardians will be asked to provide their names and contact details. Interested parents will be contacted at a later date and informed of the study procedures. Those that are willing to participate will be invited to the study's first formal meeting at the school grounds (or at an altternate suitable venue) where study procedures will be presented in detail and written consent obtained from those willing to consent to participate in study procedures.

#### Implementation of the adapted FMP program:

This will be delivered by a certified FMP trainer through six 2-hours' sessions; five sessions will be administered over a 5-week period and the 6th will be conducted six months after the 5th session. The first 5 sessions will involve (i) getting to know you and understand your child, (ii) effective parenting, (iii) parents' role in sexuality education; the information in this session will also include HPV vaccination and MC for HPV prevention, (iv) how to increase your comfort and skills in discussing sexuality issues and (v) discussing sexuality and handling peer pressure. In the 6th session, (vi) a booster session will be done to review the FMP. This will be done with parents who participated in the school health program whether or not they accepted to vaccinate their daughters or allow their sons to undergo VMMC. Minutes of each of the meetings will be documented for analysis.^18^

#### Intervention Provision:

Vaccine administration and VMMC services will be availed for free at a selected public health facility for a 7 months' period from the beginning of FMP implementation. The pre/early -adolescents that undergo any procedures at the selected facility will be linked to the study by the use of study-specific referral forms and unique study-specific identifiers.

### Data Collection

Parental-child dyads will be assigned dyad-specific interlinked participant identifiers. E.g. if a parent is FMP-1-0; his or her child will be identified as FMP-1-1. Our study will therefore be able to link parent and child information to uptake. The same identifiers will be noted on the vouchers which participants will be requested to present to a selected health facility should they take up the VMMC/HPV vaccination interventions. Data will be collected from parents and their pre/early adolescent children separately using, paper or electronic data collection tools.

Data from parents/guardians will include demographic information i.e. age, sex, relationship to the child, education level, marital status, occupation, number and ages of other children, communication with their children about sexual matters, knowledge about HIV and cervical cancer, participation in cervical cancer screening programs for females and VMMC programs from males, whether they are from a traditionally-circumcising or non-circumcising community, etc. Data from the adolescents will include demographic information, e.g. age, sex, education level, knowledge on HIV prevention and source of that knowledge, communication with their parent about sexual matters, etc. Data will be collected at baseline (prior to the 1st session) and after completion of the 5th session. During each session, anonymised deliberations will be documented and analysed to document experiences of the participants. Data will be entered into an MS Access Database and uploaded into SAS 9.2 for analysis.^24^

### Data Analysis

Measures of central tendency and proportions will be used to summarise characteristics of parent child-dyads. One parent-child dyad will be considered as one unit. HPV vaccine up-take will be described as the proportion of eligible adolescent girls who received the HPV vaccination during the study period. Among those who received the initial dose of HPV vaccine, HPV completion will be described as having received all the 3 doses of HPV. Logistic regression will be used to describe parental and child characteristics associated with HPV vaccine uptake and completion. VMMC uptake will be described as the proportion of eligible pre/early-adolescent boys who underwent VMMC during the study period. Logistic regression will be used to describe parental and child characteristics associated with VMMC uptake.^20^ All assumptions regarding type, independence, normality and variance of the data will be tested.^25^ Thematic content analysis will be used to identify emerging themes and document barriers and facilitators to promoting HPV vaccination and VMMC through FMP from project documents, deliberations of the sessions and study team members' experiences.^26^

### Project Timeline, Monitoring and Evaluation

**FIGURE 1.**
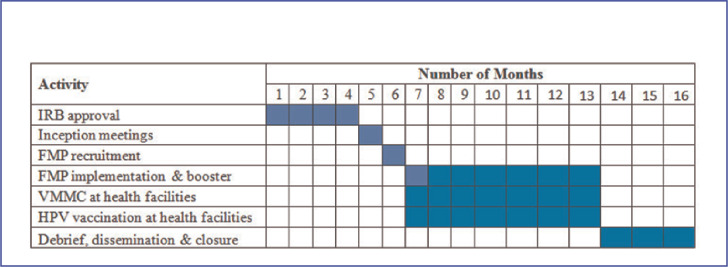
Gantt Chart For Study Implementation

This will take into consideration the length of the FMP program, i.e., 5 weeks for implementing the intervention and a booster session 6 months later.^18^ HPV vaccination is also given over a 6 months' period.^8^ Moreover, HPV and VMMC services will be available for free at selected public health facilities for a 7 months period from the beginning of the FMP sessions. The study timeline is shown in figure 1. Implementation lessons will be documented and shared during debrief sessions and later prepared for publication.

### Ethical Considerations

Written informed consent will be obtained from parents/guardians of the participants and assent from the minors. The informed consent process will be administered in the language the participants and guardians feels most comfortable, in English, Kiswahili or Dholuo. They will also be informed of the referral procedures as described in the intervention. A detailed description of all procedures including period of study will be provided.

No personal identifiers will be collected and all collected information shall remain private. All data collected will be kept secured, in locked storage spaces or in password-protected files and computers for the digital files. Furthermore, all staff will be trained in ethical procedures and measures to protect confidentiality; they will also be required to sign confidentiality agreements. Ethical approval for the conduct of this study will be sought and obtained from the Jaramogi Oginga Odinga Teaching and Referral Hospital (JOOTRH) Ethics Review Committee that cater for research projects in Kisumu County. Study approval will only commence following liaison and approval with the County government of Kisumu, officials from the ministry of education and the heads of the participating school and health facility.

#### Dissemination of study findings:

Study findings will be disseminated internally to participants in a debrief session, staff of the participating schools, county government officials and externally in national stakeholder forums, international conferences and in peer-reviewed publications.

#### Expected application of results:

**FIGURE 2.**
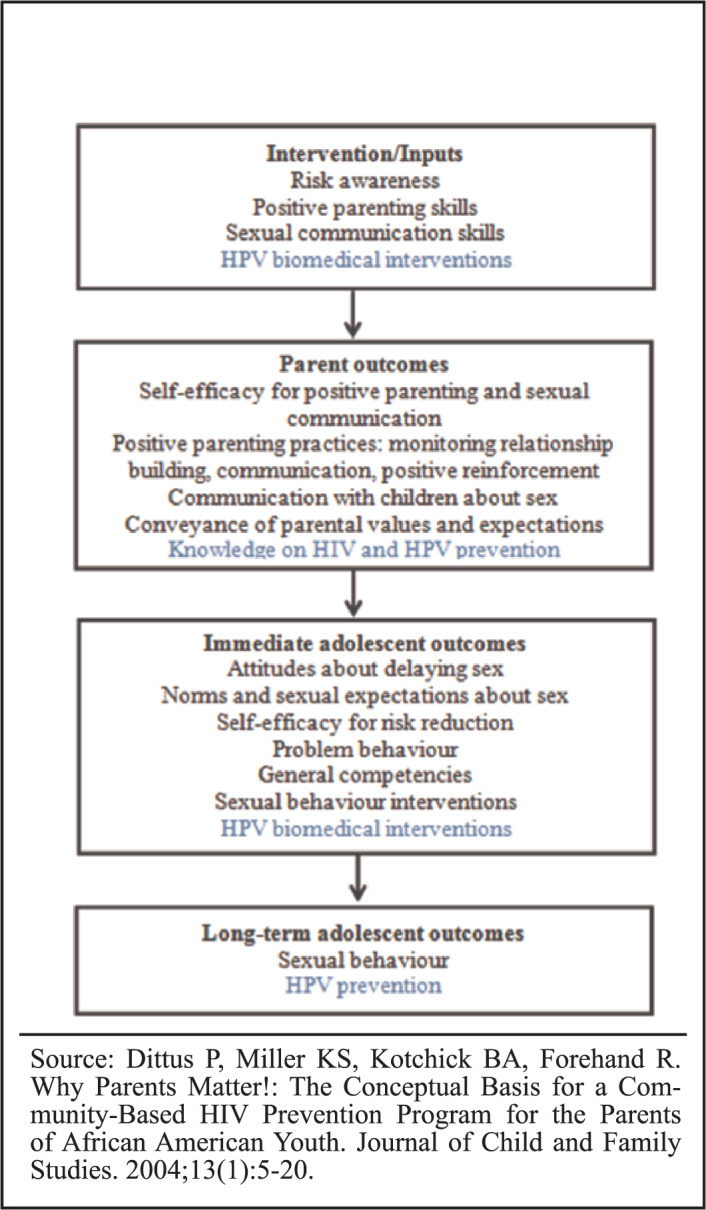
Inputs and Outputs of the Adapted FMP Program

Source: Dittus P, Miller KS, Kotchick BA, Forehand R. Why Parents Matter!: The Conceptual Basis for a Community-Based HIV Prevention Program for the Parents of African American Youth. Journal of Child and Family Studies. 2004;13(1):5-20.

It is anticipated that the intervention will enhance parental-child communication about sexual matters, promote safe sexual practices and overall reproductive health among the pre/early-adolescents and the uptake of biomedical HIV and HPV prevention interventions. Figure 2 shows the proposed inputs and expected outputs of the adapted FMP (black font) that includes HPV prevention (blue font).^27^
